# The Harvest of the Sick Poor

**Published:** 1892-01-16

**Authors:** 


### PASSING TOPICS.

### THE HARVEST OF THE SICK POOR.

In the hospital world, the locality of "home" is decidedly antipodean. Sickness, as we well know, has often times a great deal to do with turning things topsy-turvy even in the best regulated households, and it is quite in the natural order of events in a philanthropic planet that Chrismastide and the early days of the new year should be the period of harvest. Of course phenomenal fruition may occur at any time. We have known astute secretaries to make very good hay out of season, while fortunate ones have found themselves in clover all the year round. By means of festivals abundant crops may be forced at that most unpropitious of all times—an English spring, when the nipping influences of the East wind are successfully overcome by a liberal dressing of the parched soil, and the genial persuasion of a luminary of sufficient magnitude. But, as a rule, conservative and old-fashioned notions prevail. They are dear to the heart of charity who never takes kindly to innovations, and with commendable regularity and unwavering faith, she proceeds to work under the somewhat uninviting auspices of a winter sun.

And now the time has come when we may ask, what are the results of the labours which have been so abundant during the past month? Have the appeals which have followed the benevolent with so unfailing constancy met with a reception such as has satisfied the senders and assured to their institutions a year of plenty? We are something more than doubtful. Rumours reach us of failure and disappointment on the part of some of our most deserving institutions, and we search in vain for evidence that the crying need of our hospitals has received the serious attention of the community.

Time was when it was believed that a deserving work never lacked support. We fear experience teaches us that notwithstanding the wonderful vitality of every really good undertaking, and its ability to survive adverse circumstances, there is no justification for indulging in a comfortable confidence that its promoters will be spared their full share of vicissitude and discouragement. Such public feeling as can be brought to bear upon the matter is difficult to arouse, while the sympathy of many people who are undoubtedly well meaning and benevolent is very easily alienated.

A great responsibility rests upon those who seek to arrest the flow of necessary assistance, yet how lightly it is assumed. Incompetent and ignorant, or only half informed, fault-finders have much to answer for, and all the more because, unfortunately, an ounce of complaint is allowed to outweigh a ton of praise. It is this curious fact that renders so much more arduous and anxious the task of supporting every undertaking dependent upon public good-will. One gentleman pleasantly hibernating at Cannes writes to the papers to dogmatise about the "unnecessary extravagance" of the Metropolitan hospitals, inasmuch as they spend more per bed than provincial institutions. Were we in a position to refute this writer's contention utterly, we could not hope to undo the mischief caused by the mere statement. Those who understand the different conditions under which the London hospitals labour would not be surprised that they should spend more, but by the mere fact that the comparison has been instituted the hospitals are the losers, and the end is attained which, we suppose, this gentleman set before himself when he was at the pains to indite his letter. Admitting that, we may well ask, *cui bono?* Agricola.

